# A light-powered self-rotating liquid crystal elastomer drill

**DOI:** 10.1016/j.heliyon.2024.e27748

**Published:** 2024-03-15

**Authors:** Yong Yu, Haoyu Hu, Haiyang Wu, Yuntong Dai, Kai Li

**Affiliations:** School of Civil Engineering, Anhui Jianzhu University, Hefei 230601, China

**Keywords:** Drill, Self-rotation, Liquid crystal elastomer, Light-powered, Fiber

## Abstract

Self-oscillating systems can directly convert ambient energy to mechanical work, and new type self-oscillating systems are worth designing for applications in energy harvesters, engines, and actuators. Taking inspiration from the hand drill, we have developed a novel self-rotating drill system, which is consist of a turnplate and a liquid crystal elastomer (LCE) fiber under steady illumination. To investigate the self-rotating behaviors of the LCE drill, we have proposed a nonlinear theoretical model of the LCE drill under steady illumination based on the well-established dynamic LCE model. Numerical calculation reveals that the LCE drill can undergo a supercritical Hopf bifurcation between the static regime and the self-rotation regime. The self-rotation of drill originates from the contraction of winding portion of LCE fiber in illumination at winding state, and its continuous periodic motion is sustained by the interrelation between light energy and damping dissipation. The Hopf bifurcation conditions are also investigated in detail, as well as the vital system parameters affecting its frequency and amplitude. In contrast to the abundant existing self-oscillating systems, this self-rotating drill stands out due to its simple and lightweight structure, customizable dimensions, and high speed, and thus facilitates the design of compact and integrated systems, enhancing their applicability in microdevices and systems. This bears great significance in fields like micro-robotics, micro-sensors, and medical instruments, enabling the realization of smaller and higher-performance devices.

## 1 Introduction

Self-sustained oscillation is a periodic oscillation phenomenon triggered by external steady excitations [1]. Self-oscillation can be maintained in a continuous periodic manner by means of periodically absorbing energy from constant external stimuli, without the need for additional complex controllers and portable batteries [[2], [3], [4], [5], [6]]. Thus, to a certain extent, the complexity of self-oscillating systems is reduced, and interesting applications including portability become possible [[7], [8], [9], [10], [11], [12], [13]]. In addition, the period and amplitude of self-oscillation generally depend on the parameters of the system itself and are almost independent of the initial conditions, which makes the system robust [14,15]. Taking advantage of these superiorities in self-oscillation, self-oscillating systems are becoming attractive candidates for a variety of applications, including active machines [[16], [17], [18], [19], [20], [21], [22]], autonomous robotics [23], energy-absorbing devices [[24], [25], [26]], motors [27], etc.

Based on stimuli-responsive materials, including liquid crystal elastomers (LCEs) [28], ionic gels [29,30], hydrogels [31,32], etc., diverse self-oscillating systems have been widely developed recently. Especially, there have been numerous attempts to construct a large number of self-sustained motion patterns, such as vibration [33], bending [34,35], rolling [[36], [37], [38]], spinning [39], torsion [40], shuttling [41], self-oscillating auxetic metamaterials [42], self-floating [43] and self-curling [44], shrinking [45], swimming [46], swinging [16,47], buckling [48,49], jumping [50,51], rotation [52,53], chaos [54] and even synchronized motion of coupled self-oscillators [55]. In these self-oscillating systems, some special mechanisms are generally required for absorbing energy from the external environment to compensate for the dissipation consumed by the system damping [1]. Based on different stimuli-responsive materials, various feedback mechanisms have been proposed to achieve energy compensation [16,17,56], such as coupling mechanism between chemical reaction and large deformation [29,30], self-shading mechanism [16,17,57], and multi-process coupling mechanism between droplet evaporation and movement [58]. These mechanisms are always accompanied by nonlinear coupling of multiple processes and self-oscillatory feedback [[56], [57], [58]].

Among the stimuli-responsive materials that constitute the self-excited oscillatory system, LCE has some special advantages. LCE is fabricated by synthesizing anisotropic rod-like liquid crystal molecules and stretchable long-chain polymers [59]. When exposed to environmental stimuli, such as light [21], heat [60], electricity [61] and magnetism [62], the liquid crystal monomer molecules would rotate or undergo phase transitions, to change their configurations, thereby generating macroscopic deformation [63]. Among these external stimuli, optical stimulation is ideal because it allows for remote and noncontact operation. Meanwhile, convenient and precise adjustments can be made in terms of intensity, wavelength, and polarization directions. In addition, optical excitation offers appealing features of abundant and clean light sources. Meanwhile, the optically-responsive LCE exhibits fast response, large intrinsic deformation as well as reversible deformation. These special features allow the induce of feedback in various approaches, resulting in light-induced self-sustained oscillations [64], which in turn has led to the extensive use of LCE-based light-fueled self-oscillating systems [[63], [64], [65]].

**Fig. 1:**
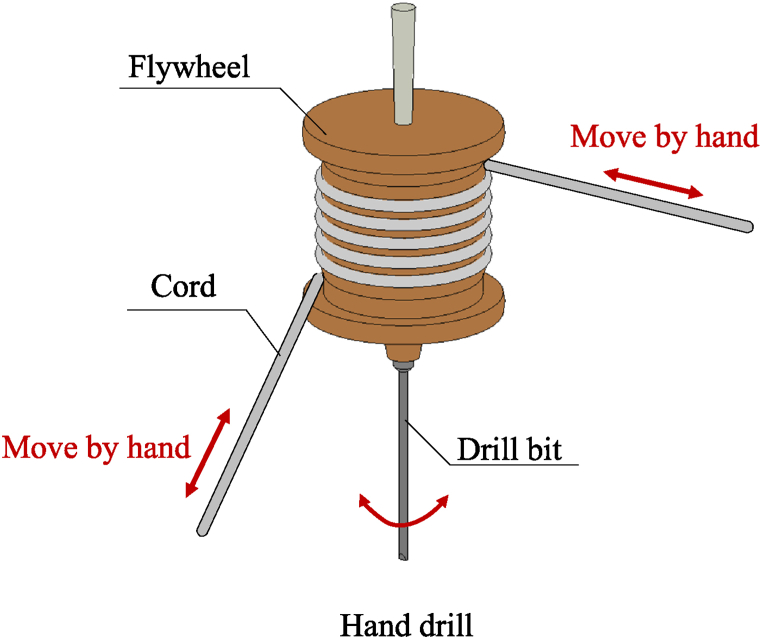
Schematic diagram of a hand drill, which mainly consists of a cord, flywheel, and drill bit. The rotation of the flywheel and drill bit is achieved by rhythmically controlling the movement of the cord.

Despite the widespread interest in LCE-based self-oscillating systems has led to the construction of many self-oscillating systems, there is still a need for building self-exciting systems with more diverse motion patterns in order to meet the demands of various functional applications. The hand drill is a conventional device that is easy to construct and mainly consists of a cord, flywheel, and drill bit. It can effectively transform rhythmic translational motions into vibratory, bi-directional rotary insertions. One can rhythmically control the translational motion of cord to drive the rotation of the flywheel and drill bit, as shown in Fig. 1. Inspired by the hand drill, we innovatively propose a new type of self-rotating drill, which consists of a turnplate and an LCE fiber. Under steady illumination, the LCE drill can rotate continuously and bidirectionally. The objective of this paper is to construct a light-powered self-rotating LCE drill, investigate its dynamic behaviors, and provide guide for its applications in engineering. The current industrial drill has the disadvantages of large volume, heavy and complex structure. Meanwhile, it usually involves complex parameter adjustment and complex integration process, and the practicability is low. The proposed self-rotating system has several unique advantages such as simple structure, customizable size, fast speed, light weight and robust [[66], [67], [68]], and enables miniaturization and integration, making it more convenient for application in micro-devices and systems. This would be of significant importance in fields such as micro-robotics, micro-sensors, and medical instruments, enabling the realization of smaller and higher-performance devices.

The remainder of this paper is structured as follows. In Section 2, we first propose a nonlinear dynamic model of self-rotating drill under steady illumination, involving the well-established dynamic LCE model, and then formulate the corresponding governing equations. Section 3 presents two motion regimes of the LCE drill under steady illumination, and discusses the mechanism of self-rotation in detail. In Section 4, we focus on the Hopf bifurcation conditions between the static and self-rotation regimes and the influences of various system parameters on its frequency and amplitude. Finally, we conclude our study in Section 5.

## 2 Model and formulation

In this section, we first propose a self-rotating LCE drill under steady illumination upon inspiration of hand drill as shown in Fig. 1. The LCE drill consists of a LCE fiber and a turnplate, and the turnplate can rotate continuously and bidirectionally under steady illumination. Based on dynamic LCE model, a dynamic model for the self-rotation of LCE drill under steady illumination is established. The main contents include the rotational dynamics of the self-rotating drill, the light-driven rotational moment, the tension of LCE fiber, the evolution of the number of *cis-isomers* in LCE fiber, nondimensionalization, and the solution method of the differential governing equations with variable coefficients.

### 2.1 Dynamics of the LCE drill

**Fig. 2:**
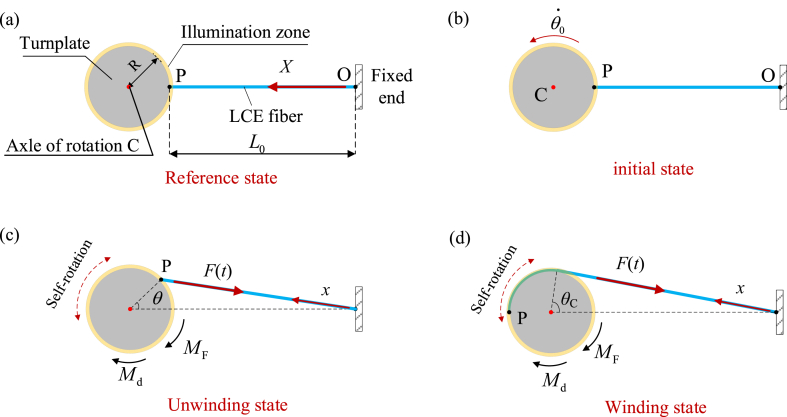
Schematics of a self-rotating drill, which consists of a LCE fiber and a turnplate: (a) reference state, (b) initial state, (c) unwinding state, (d) winding state. The narrow area Δ around the turnplate is steadily illuminated. The LCE drill can maintain a continuous periodic rotation under the steady illumination.

Fig. 2 sketches the self-rotating LCE drill, which consists of a LCE fiber and a turnplate. We set the initial length of LCE fiber $L_{0}$ to be 0.2 m and the radius R of the turnplate to be 0.002 m. The ratio between R and $L_{0}$ is approximately 1%, and the deformation in the self-rotation is less than 10%. The narrow area Δ around the turnplate is steadily illuminated. The light is selected for parallel UV or laser with wavelength of 360 nm [69]. Under the illumination, the *trans-to-cis* isomerization of LCE could be induced. One end of the LCE fiber is connected to the fixed end O, and the other end is connected to point P on the circular turnplate with radius *R* and an rotating axel C. In the reference state, the LCE fiber is stress-free with an original length of $L_{0}$, and the azobenzene liquid crystal molecules in the fiber is in the straight *trans* state and along its longitudinal direction (Fig. 2 (a)). Initially, the LCE drill is given an initial angular velocity $\overset{\cdot }{\theta_{0}}$ from the reference state, and the turnplate starts to rotate (Fig. 2(b)). At the start of the rotation, the LCE fiber keeps straight and does not wind around the turnplate, which is named as the unwinding state (Fig. 2 (c)). In the unwinding state, the LCE fiber is not yet in the illuminated zone Δ and the azobenzene liquid crystal molecules in the LCE fiber stay in straight *trans* state. Then, as the turnplate rotates to the position with critical rotational angle $\theta_{c}=\arccos\frac{R}{R+L_{0}}$, the LCE fiber starts to wind around the turnplate (Fig. 2 (d)). The winding portion of the LCE fiber is illuminated and contracts, for the corresponding azobenzene liquid crystal molecules transform from a straight *trans* state to a bent *cis* state under illumination.

The light-driven contraction increases the tension F(t) in the fiber and the corresponding rotational moment $M_{F}\left(t\right)$, which always tends to rotate the turnplate back into the equilibrium position. As the rotation angle of the turnplate increases, the winding portion exposed to light gradually increases, and the tension F(t) also increases significantly as the winding portion increases, eventually decelerating the turnplate rotation and driving the turnplate to rotate in the opposite direction. Subsequently, as the turnplate rotates in the opposite direction, the LCE fiber moves from the winding state to unwinding state. In the unwinding state, the light-driven contraction and tension F(t) decreases due to the transformation of the corresponding azobenzene liquid crystal molecules from a bent *cis* state to a straight *trans* state. Because of its own inertia, the rotating turnplate then passes through the equilibrium position and continues to rotate, which makes the LCE fiber wind around the turnplate in the opposite direction. Finally, under the steady illumination, the turnplate may exhibit a continuous periodic rotation.

As shown in Fig. 2, the turnplate is subjected to the rotational moment $M_{F}\left(t\right)$ and the damping moment $M_{d}\left(t\right)$. For simplicity, we assume that the damping moment $M_{d}\left(t\right)$ is proportional to its rotation angular velocity. The corresponding nonlinear dynamic governing equation of the self-rotating drill can be derived as

(1) $$J\frac{d^{2}\theta \left(t\right)}{dt^{2}}=M_{F}\left(t\right)-\beta \frac{d\theta \left(t\right)}{dt}\text{,}$$

where β is the damping coefficient and *J* refers to the moment of inertia of the turnplate about its center point C. In addition, the initial condition is given as

(2) $$\theta \left(t\right)=\theta_{0},\frac{d\theta \left(t\right)}{dt}=\overset{\cdot }{\theta_{0}}\;\text{at}\;t=0\text{.}$$

Noted that the rotational moment $M_{F}\left(t\right)$ in Eq. (1) results from the tension of LCE fiber, which will be calculated in the following.

### 2.2 Rotational moment

The rotational moment $M_{F}\left(t\right)$ can be determined by the tension F(t) of the LCE fiber. For the two cases of unwinding state ($\left|\theta \right|\leq \theta_{C}$) and winding state ($\left|\theta \right|>\theta_{C}$) in the process of drill rotating, the rotational moment $M_{F}\left(t\right)$ has different analytical expressions. To simplify the formulation, four assumptions are made: 1. The mass of LCE fiber is negligible; 2. The cross-sectional shape of LCE fiber remains constant under tension; 3. Friction between LCE fiber is ignored; 4. Tension is uniform throughout the entire LCE fiber.

#### 2.2.1 Unwinding state

For the unwinding state, i.e., $\left|\theta \right|\leq \theta_{C}$, as shown in Fig. 2 (c), the rotational moment $M_{F}\left(t\right)$ can be easily calculated as follows

(3) $$M_{F}\left(t\right)=-\left(L_{0}+R\right)R\;\sin\;\theta \frac{F\left(t\right)}{L\left(t\right)}\text{sgn}\left(\theta \right)\text{,}$$

where sgn(θ) equals to θ/|θ| when θ≠0, and zero otherwise. The current LCE fiber length L(t) in unwinding state can be easily obtained form Fig. 2 (c) as

(4) $$L\left(t\right)=\sqrt{R^{2}+{\left(L_{0}+R\right)}^{2}-2R\left(R+L_{0}\right)\cos\;\theta }\text{.}$$

#### 2.2.2 Winding state

For the winding state, i.e., $\left|\theta \right|>\theta_{C}$, the rotational moment $M_{F}\left(t\right)$ is closely related to the tension F(t). As shown in Fig. 2 (d), the tension direction of the fiber is along its center line. Referring to the mature model of rigid rotation about a fixed axis [70,71], the calculation formula for the rotational moment $M_{F}\left(t\right)$ in the winding state is as follows

(5) $$M_{F}\left(t\right)=-F\left(t\right)R\text{sgn}\left(\theta \right)\text{,}$$

and the current LCE fiber length L(t) in winding state can be easily obtained from Fig. 2 (d) as

(6) $$L\left(t\right)=\sqrt{{\left(L_{0}+R\right)}^{2}-R^{2}}+R\left(\left|\theta \right|-\theta_{C}\right)\text{.}$$

### 2.3 Tension of the LCE fiber

In order to determine the rotational moment $M_{F}\left(t\right)$ in Eqs. (3), (5), it is necessary to calculate the tension F(t). For simplicity, the friction and mass of the fiber are ignored in the calculation, the tension F(t) of the fiber is assumed to be proportional to the elastic strain $\varepsilon_{e}$, i.e.

(7) $$F\left(t\right)=kL_{0}\varepsilon_{e}\left(t\right)\text{,}$$

where *k* is the spring constant of LCE fiber. It is worth noting that $\varepsilon_{e}\left(t\right)$ is homogeneous in the LCE fiber, whereas both the total strain $\varepsilon_{\text{tot}}$ and the light-driven contraction $\varepsilon_{L}$ are inhomogeneous. To analyze the inhomogeneous deformation of the LCE fibers, we introduce the Lagrangian arc coordinate system *X* in the reference state (Fig. 2 (a)) and the Eulerian arc coordinate system *x* in the current state (Fig. 2 (c)). During the rotation of turnplate, the instantaneous position of a material point in the LCE fiber can be described as *x* = *x* (*X*,*t*). Then, the total strain and the light-driven contraction in LCE fiber can be expressed as $\varepsilon_{\text{tot}}\left(X,t\right)$ and $\varepsilon_{L}\left(X,t\right)$, respectively. For simplicity, the elastic strain in LCE fiber $\varepsilon_{e}\left(t\right)$ under small deformation can be assumed as a linear combination of the total strain $\varepsilon_{\text{tot}}\left(X,t\right)$ and the light-driven contraction $\varepsilon_{L}\left(X,t\right)$, i.e. $\varepsilon_{e}\left(t\right)=\varepsilon_{\text{tot}}\left(X,t\right)-\varepsilon_{L}\left(X,t\right)$. Thus, the tension can be rewritten as

(8) $$F\left(t\right)=kL_{0}\left[\varepsilon_{\text{tot}}\left(X,t\right)-\varepsilon_{L}\left(X,t\right)\right]\text{,}$$

where $\varepsilon_{L}\left(X,t\right)$ is assumed to be a linear function of the number fraction of *cis-isomers* φ(X,t), i.e.

(9) $$\varepsilon_{L}\left(X,t\right)=-C_{0}\varphi \left(X,t\right)\text{,}$$

where $C_{0}$ is the contraction coefficient.

For simplicity, the total strain is defined as $\varepsilon_{\text{tot}}\left(X,t\right)=\lambda \left(X,t\right)-1=\frac{dx-dX}{dX}$, where the deformation gradient λ(X,t) is defined as

(10) $$\lambda \left(X,t\right)=\frac{dx\left(X,t\right)}{dX}\text{.}$$

Thus, F(t) in Eq. (8) can be rewritten as

(11) $$F\left(t\right)=kL_{0}\left[\lambda \left(X,t\right)-1+C_{0}\varphi \left(X,t\right)\right]\text{.}$$

By integrating Eq. (11) from 0 to $L_{0}$ and dividing both sides of this equation by $L_{0}$, we can obtain

(12) $$F\left(t\right)=k\left[L\left(t\right)-L_{0}+C_{0}\int_{0}^{L_{0}}\varphi \left(X,t\right)dX\right]\text{.}$$

### 2.4 Dynamic LCE model

To calculate the number fraction φ(X,t) of the *cis-isomers* of LCE fiber in Eq. (12), the well-established dynamic LCE model proposed by Finkelmann et al. is utilized [59,72]. Found by Yu et al. that the *trans-to-cis* isomerization of LCE could be induced by UV or laser with wavelength about 360 nm [69]. The number fraction φ(X,t) of the *cis-isomers* depends on the thermal excitation from *trans to cis*, the thermally driven relaxation from *cis to trans*, and the light driven relaxation from *trans to cis*. The thermal excitation from *trans to cis* is often considered negligible relative to the light-driven excitation [72,73], thereby, the number fraction φ(X,t) can be usually described by the following governing equation

(13) $$\frac{\partial \varphi \left(X,t\right)}{\partial t}=\eta_{0}I\left(X,t\right)\left[1-\varphi \left(X,t\right)\right]-\tau_{0}^{-1}\varphi \left(X,t\right)\text{,}$$

where $\eta_{0}$ is the light absorption constant and $\tau_{0}$ is the thermal relaxation time from *cis to trans.*

To solve Eq. (13), the current position x(X,t) should be calculated to judge whether the material point *X* is in illumination or darkness. Combining Eqs. (11), (13), λ(X,t) can be written as

(14) $$\lambda \left(X,t\right)=\frac{1}{L_{0}}\left[L\left(t\right)-L_{0}+C_{0}\int_{0}^{L_{0}}\varphi \left(X,t\right)dX\right]+1-C_{0}\varphi \left(X,t\right)\text{.}$$

From Eqs. (10), (14), we can obtain

(15) $$dx\left(X,t\right)=\left\{\frac{1}{L_{0}}\left[L\left(t\right)-L_{0}+C_{0}\int_{0}^{L_{0}}\varphi \left(X,t\right)dX\right]+1-C_{0}\varphi \left(X,t\right)\right\}dX\text{.}$$

By integrating both sides of Eq. (15) from 0 to X and considering that x(0,t)=0, we have

(16) $$x\left(X,t\right)=\frac{X}{L_{0}}\left[L\left(t\right)-L_{0}+C_{0}\int_{0}^{L_{0}}\varphi \left(X,t\right)dX\right]+X-C_{0}\int_{0}^{X}\varphi \left(X,t\right)dX\text{.}$$

### 2.5 Nondimensionalization

For convenience, we introduce the dimensionless quantities as follows: $\bar{t}=t/\tau_{0}$, $\bar{L}=L/L_{0}$, $\bar{R}=R/L_{0}$, $\bar{X}=X/L_{0}$, $\bar{x}=x/L_{0}$, ${\bar{M}}_{F}=M_{F}\tau_{0}^{2}/J$, $\bar{F}=FL_{0}\tau_{0}^{2}/J$, $\bar{\beta }=\beta \tau_{0}/J$, $\bar{k}=k\tau_{0}^{2}L_{0}^{2}/J$, $\bar{I}=I\eta_{0}\tau_{0}$, ${\bar{I}}_{0}=I_{0}\eta_{0}\tau_{0}$. Eqs. (1), (2), (3), (4), (5), (6), (12), (13), (16) can be rewritten as follows

(17) $$\frac{d^{2}\theta \left(\bar{t}\right)}{d{\bar{t}}^{2}}={\bar{M}}_{F}\left(\bar{t}\right)-\bar{\beta }\frac{d\theta \left(\bar{t}\right)}{d\bar{t}}\text{,}$$

(18) $$\theta \left(\bar{t}\right)={\bar{\theta }}_{0},\frac{d\theta \left(\bar{t}\right)}{dt}={\overset{\cdot }{\bar{\theta }}}_{0}\;\text{at}\;\bar{t}=0\text{,}$$

where ${\bar{M}}_{F}\left(\bar{t}\right)$ can be calculated as,

For the unwinding state ($\left|\theta \right|\leq \theta_{C}$),

(19) $${\bar{M}}_{F}\left(\bar{t}\right)=-\left(1+\bar{R}\right)\bar{R}\sin\;\theta \frac{\bar{F}\left(\bar{t}\right)}{\bar{L}\left(\bar{t}\right)}\text{sgn}\left(\theta \right)\text{,}$$

where the current LCE fiber length $\bar{L}\left(\bar{t}\right)$ in unwinding state can be easily calculated as

(20) $$\bar{L}\left(\bar{t}\right)=\sqrt{{\bar{R}}^{2}+{\left(1+\bar{R}\right)}^{2}-2\bar{R}\left(\bar{R}+1\right)\cos\;\theta }\text{,}$$

and for the winding state ($\left|\theta \right|>\theta_{C}$),

(21) $${\bar{M}}_{F}\left(\bar{t}\right)=-\bar{F}\left(\bar{t}\right)\bar{R}\text{sgn}\left(\theta \right)\text{,}$$

with LCE fiber length $\bar{L}\left(\bar{t}\right)$ in winding state is

(22) $$\bar{L}\left(\bar{t}\right)=\sqrt{{\left(1+\bar{R}\right)}^{2}-{\bar{R}}^{2}}+\bar{R}\left(\left|\theta \right|-\theta_{C}\right)\text{,}$$

where $\bar{F}\left(\bar{t}\right)$ is

(23) $$\bar{F}\left(\bar{t}\right)=\bar{k}\left[\bar{L}\left(\bar{t}\right)-1+C_{0}\int_{0}^{1}\varphi \left(\bar{X},\bar{t}\right)d\bar{X}\right]\text{,}$$

and $\varphi \left(\bar{X},\bar{t}\right)$ can be calculated as

(24) $$\frac{\partial \varphi \left(\bar{X},\bar{t}\right)}{\partial \bar{t}}=\bar{I}\left(\bar{X},\bar{t}\right)\left[1-\varphi \left(\bar{X},\bar{t}\right)\right]-\varphi \left(\bar{X},\bar{t}\right)\text{.}$$

The dimensionless instantaneous position $\bar{x}\left(\bar{X},\bar{t}\right)$ can be determined by follows

(25) $$\bar{x}\left(\bar{X},\bar{t}\right)=\bar{X}\left[\bar{L}\left(\bar{t}\right)-1+C_{0}\int_{0}^{1}\varphi \left(\bar{X},\bar{t}\right)d\bar{X}\right]+\bar{X}-C_{0}\int_{0}^{\bar{X}}\varphi \left(\bar{X},\bar{t}\right)d\bar{X}\text{.}$$

Eqs. (17), (24) govern the self-rotating drill with a LCE fiber under steady illumination, in which the time-dependent number fraction of *cis-isomers* is coupled to the angular position of the self-rotating drill. To solve these complex differential equations with variable coefficients, Runge-Kutta method is ulitized and numerical calculation is performed in Matlab software. Upon completion of the convergence analysis of numerical calculations, a time step of 0.002 is selected for the computation. For the number fraction $\varphi_{i}$ of *cis-isomers* and the position $\theta_{i}$ of turnplate at time ${\bar{t}}_{i}$, the current tensions ${\bar{F}}_{i}$ of LCE fiber and the current rotational moment ${\bar{M}}_{\text{Fi}}$ can be determined by Eqs. (19), (20), (21), (22), (23). Then the angular position of the turnplate $\theta_{i+1}$ at time ${\bar{t}}_{i+1}$ can be calculated using Eq. (17). Meanwhile, the current position ${\bar{x}}_{i}\left(\bar{X},{\bar{t}}_{i}\right)$ can be determined by Eq. (25) to estimate the light intensity $\bar{I}\left(\bar{X},{\bar{t}}_{i}\right)$, and then the number fraction $\varphi_{i+1}$ of *cis-isomers* in the LCE fiber can be calculated using Eq. (24).

## 3 Two motion regimes and mechanism of self-rotation

In this section, based on the solution of the governing Eqs. (17), (24), we first present two typical motion regimes of the LCE drill, which are distinguished as the static regime and the self-rotation regime. Next, the corresponding mechanism of self-rotation is investigated in detail.

### 3.1 Two motion regimes

To investigate the self-rotating LCE drill under steady illumination, we should determine the typical values of the dimensionless parameters in the model. From the available experiments [16,74,75], the typical material properties and geometric parameters are listed in Table 1. The corresponding dimensionless parameters are also listed in Table 2.

**Table 1:** Material properties and geometric parameters.

Parameter	Definition	Value	Unit
β	Damping coefficient	0∼0.001	$\text{mg}\cdot {\text{mm}}^{2}/s$
$C_{0}$	Contraction coefficient	0∼0.4	/
$\eta_{0}$	Light-absorption constant	0.0003	$m^{2}/\left(s\cdot W\right)$
$\tau_{0}$	*Trans-to-cis* thermal relaxation time	1∼100	ms
*J*	Moment inertia of turnplate	0∼1	$\text{mg}\cdot {\text{mm}}^{2}$
$I_{0}$	Light intensity	0∼65	$\text{KW}/m^{2}$
$L_{0}$	Length of LCE fiber	1	m
*k*	Spring constant	9.5	N/m
*R*	Radius of turnplate	0.01	m

**Table 2:** Dimensionless parameters.

Parameter	$\theta_{0}$	${\overset{\cdot }{\bar{\theta }}}_{0}$	$\bar{\beta }$	$\bar{I_{0}}$	$C_{0}$	$\bar{k}$	$\bar{R}$
**Value**	0∼10	0∼10	0∼0.1	0∼1.95	0∼0.4	0∼4600	0∼0.1

**Fig. 3:**
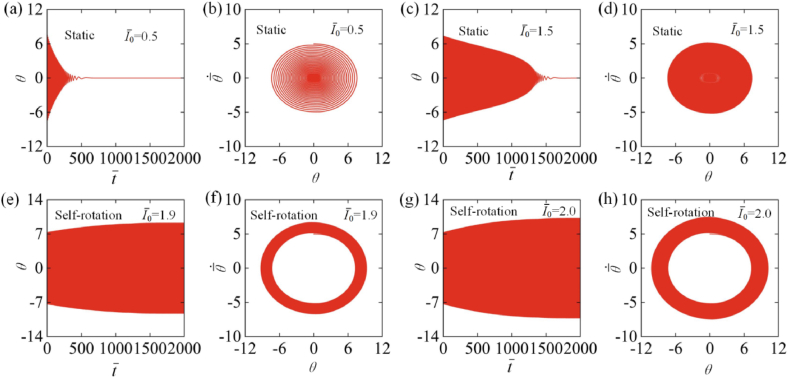
Time histories and phase trajectories for the two motion regimes of LCE drill. (a–d) are the static regimes with ${\bar{I}}_{0}=0.5$ and ${\bar{I}}_{0}=1.5$; (e–h) are the self-rotation regimes with ${\bar{I}}_{0}=1.9$ and ${\bar{I}}_{0}=2.0$. The other parameters are $C_{0}=0.34$, $\bar{k}=4600$, $\bar{\beta }=0.026$, $\theta_{0}=0$, $\bar{R}=0.01$, ${\overset{\cdot }{\bar{\theta }}}_{0}=5$. The LCE drill under steady illumination can develop into two motion regimes: the static regime and the self-rotation regime, which means that the drill undergoes a supercritical Hopf bifurcation as shown in Section 4.

By numerically solving Eqs. (17), (18), (19), (20), (21), (22), (23), (24), (25), the time histories and phase trajectories of self-rotation can be obtained, among which the cases for ${\bar{I}}_{0}=0.5$, ${\bar{I}}_{0}=1.5$, ${\bar{I}}_{0}=1.9$ and ${\bar{I}}_{0}=2.0$ are plotted in Fig. 3 and Video 1. In the calculation, we set the other parameters as $C_{0}=0.34$, $\bar{k}=4600$, $\bar{\beta }=0.026$, $\theta_{0}=0$, $\bar{R}=0.01$, and ${\overset{\cdot }{\bar{\theta }}}_{0}=5$. For ${\bar{I}}_{0}=0.5$ and ${\bar{I}}_{0}=1.5$, the amplitude of the angular position θ exhibits a gradual decrease with time due to the damping dissipation, and the LCE drill eventually attains stationary at the equilibrium position, which is named as the static regime (Figs. 3 (a), (b), (c) and (d)). For ${\bar{I}}_{0}=1.9$ and ${\bar{I}}_{0}=2.0$, the LCE drill starts to rotate from rest, and the amplitude of the angular displacement θ gradually increases with time, eventually reaching stability. Finally, under steady illumination, the LCE drill shows continuous periodic rotation, which we refer to as the self-rotation regime (Fig. 3 (e), (f), (g) and (h)). This result means that the drill undergoes a supercritical Hopf bifurcation as discussed in Section 4. In the following, we further investigate the mechanism of self-rotation in detail.

### 3.2 Mechanism of self-rotation

**Fig. 4:**
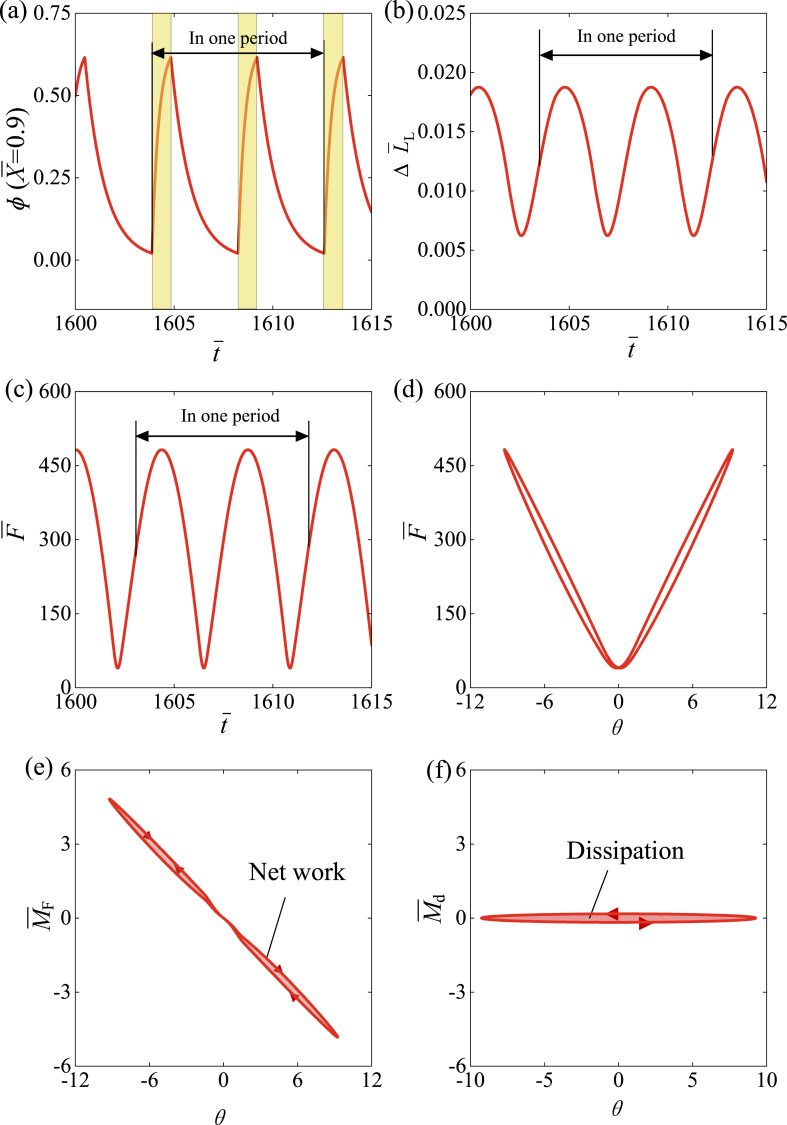
Mechanism of the self-rotation for the typical case in Figs. 3 (e) and (f). (a) The time history curve for the number fraction of *cis*-isomers at $\bar{X}=0.94$. (b) Time history curve of the purely light-induced fiber length change. (c) Time history curve of the tension of LCE fiber. (d) Relationship between tension and angular position during one cycle. (e) Relationship between rotational moment and angular position. (f) Relationship between damping moment and angular position.

**Fig. 5:**
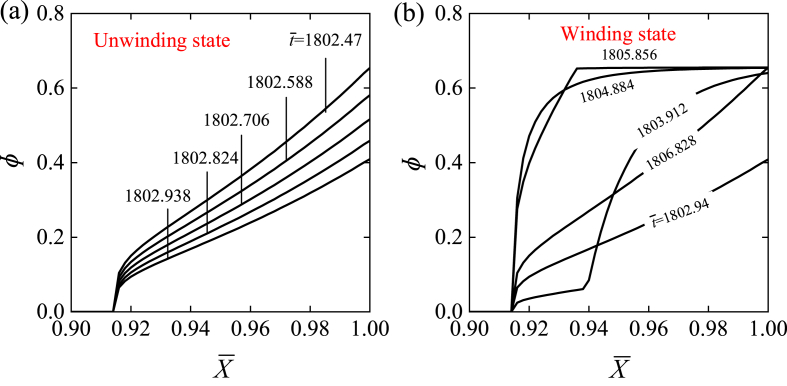
Evolution of the inhomogeneous number fraction of *cis-isomers* in LCE fiber from unwinding state to winding state for the case in Figs. 3(e) and (f). (a) Unwinding state. (b) Winding state. The number fraction of *cis*-isomers increases over time for the winding segment in illumination, while decreases for the unwinding segment in darkness.

**Fig. 6:**
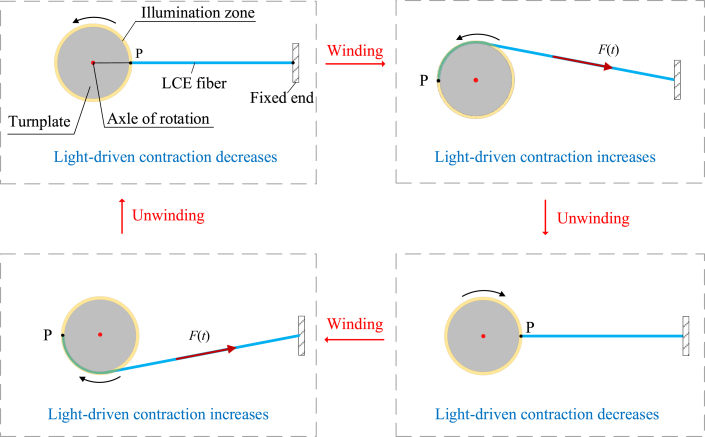
Snapshots in one cycle of self-rotation of the LCE drill under the conditions of Figs. 3 (e) and (f). Under steady illumination, the self-rotating LCE drill will exhibit a continuous periodic rotation.

To investigate the mechanism of self-rotation, Fig. 4 illustrates several important physical quantities of the LCE drill for the typical case shown in Figs. 3 (e) and (f). Fig. 4 (a) gives the time history curve of the number fraction of *cis-isomers* φ at the representative material point $\bar{X}=0.94$ of LCE fiber, reflecting the periodic variation with time. As the material enters the illuminated zone, φ gradually increases and approaches a maximum, while φ decreases gradually in the dark. Figs. 5 (a) and (b) further illustrate the distribution of the non-uniform number fraction of *cis-isomers* in the LCE fiber for the unwinding and winding states, respectively, during one cycle of the rotation. It is shown that the number fraction of *cis-isomers* increases over time for the winding segment in illumination, while decreases for the unwinding segment in darkness. This can be further understood from Fig. 6. Fig. 4 (b) and (c) show the time history curves of fiber length $\Delta {\bar{L}}_{L}=C_{0}\int_{0}^{1}\varphi \left(\bar{X},\bar{t}\right)d\bar{X}$ and tension $\bar{F}$ of LCE fiber induced purely by light, both of which exhibit periodic behaviors due to the periodic changes in the number fraction of *cis-isomers* φ.

Fig. 4 (d) further describes the relationship between tension $\bar{F}$ and angular position θ in the case of Fig. 3 (e) and (f). During forward rotation, the increase in tension $\bar{F}$ is due to the light-driven contraction and the increase in LCE fiber length. During backward rotation, tension $\bar{F}$ decreases monotonically due to the decrease in light-driven contraction and LCE fiber length. Fig. 4 (e) further reveals the relationship between rotational moment ${\bar{M}}_{F}$ and angular position θ, which is jointly determined by tension $\bar{F}$ and angular position θ, as shown by Eqs. (19), (21). The area enclosed by the curve in Fig. 4 (e) represents the positive net work done by the LCE fiber, which is numerically calculated to be 5.0278. Fig. 4 (f) shows the relationship between damping moment ${\bar{M}}_{d}$ and angular position θ, and the area enclosed by the curve in Fig. 4 (f) represents the damping dissipation, which is numerically calculated to be 5.0278. The net work done by LCE fiber is exactly identical to the dissipation, which means that the self-rotation results from the competition between the net work done by LCE fiber and damping dissipation.

## 4 Influences of system parameters on the self-rotation

In the mechanical model of self-rotation described above, there are six dimensionless system parameters including $C_{0}$, $\bar{k}$, ${\bar{I}}_{0}$, $\bar{\beta }$, $\bar{R}$, ${\overset{\cdot }{\bar{\theta }}}_{0}$. In this section, the influences of these system parameters on the Hopf bifurcation conditions, frequency and amplitude of self-rotation are studied in details.

### 4.1 Influence of light intensity

**Fig. 7:**
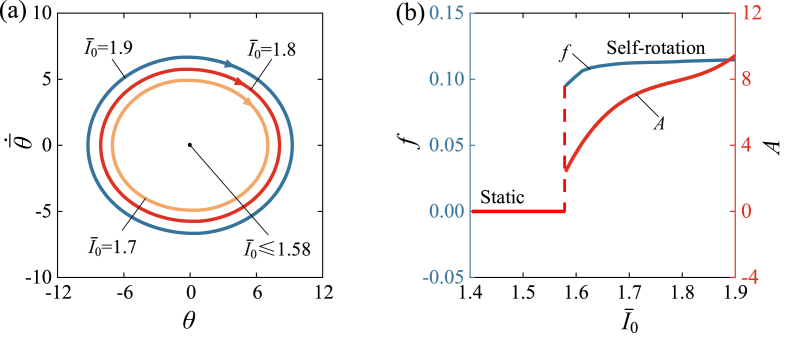
The influence of light intensity on the self-rotation, for $C_{0}=0.34$, $\bar{k}=4600$, ${\overset{\cdot }{\bar{\theta }}}_{0}=5$, $\bar{\beta }=0.026$, $\bar{R}=0.01$, ${\bar{\theta }}_{0}=0$. (a) Limit cycles. (b) Frequency and amplitude. There is a critical ${\bar{I}}_{0}\approx 1.58$ for the supercritical Hopf bifurcation between the static regime and the self-rotation regime. With the increase of ${\bar{I}}_{0}$, both the frequency and amplitude of self-rotation present upward trends.

Fig. 7 and Video 2 show the influence of light intensity ${\bar{I}}_{0}$ on the self-rotation of LCE drill. In the calculation, we set $C_{0}=0.34$, $\bar{k}=4600$, ${\overset{\cdot }{\bar{\theta }}}_{0}=5$, $\bar{\beta }=0.026$, $\bar{R}=0.01$, ${\bar{\theta }}_{0}=0$. There exists a critical value of approximately 1.58 for light intensity to trigger the self-rotation, which implies that the drill undergoes a supercritical Hopf bifurcation at the critical light intensity. When ${\bar{I}}_{0}\leq 1.58$, the self-rotating drill always stops at an equilibrium position, indicating a static regime, while the self-rotation regime can be triggered when ${\bar{I}}_{0}=1.7,1.8,1.9$. Fig. 7 (a) illustrates the limit cycles, while Fig. 7 (b) presents the influence of ${\bar{I}}_{0}$ on the self-rotating frequency and amplitude. As ${\bar{I}}_{0}$ increases, the frequency increases slightly and the amplitude increases significantly. This result can be understood from Eqs. (19), (21), (23), (24), in which ${\bar{I}}_{0}$ increases the rotational moment ${\bar{M}}_{F}$ and thus amplifies the amplitude. Self-rotation is the result of light energy being absorbed and converted into mechanical energy, and with an increase in light intensity, more energy is converted, resulting in shorter self-rotation times within one period.

### 4.2 Influence of contraction coefficient

**Fig. 8:**
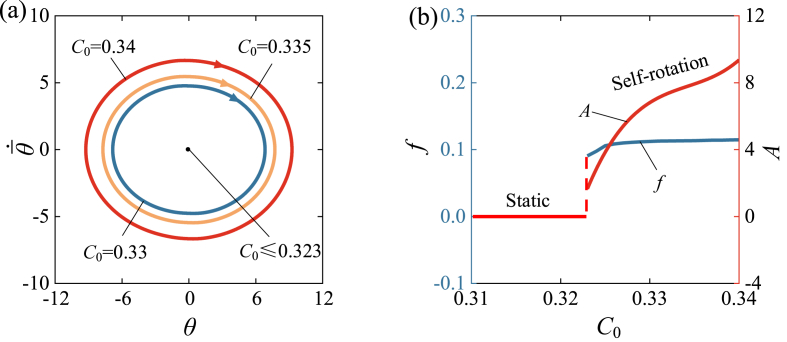
The influence of contraction coefficient on the self-rotation, for ${\bar{I}}_{0}=1.9$, $\bar{k}=4600$, ${\overset{\cdot }{\bar{\theta }}}_{0}=5$, $\bar{\beta }=0.026$, $\bar{R}=0.01$, ${\bar{\theta }}_{0}=0$. (a) Limit cycles. (b) Frequency and amplitude. There is a critical $C_{0}\approx 0.323$ for the supercritical Hopf bifurcation between the static regime and the self-rotation regime. With the increase of $C_{0}$, both the frequency and amplitude of self-rotation tend to increase.

Fig. 8 and Video 3 show the influence of contraction coefficient $C_{0}$ on the self-rotation of LCE drill. In the calculation, we set ${\bar{I}}_{0}=1.9$, $\bar{k}=4600$, ${\overset{\cdot }{\bar{\theta }}}_{0}=5$, $\bar{\beta }=0.026$, $\bar{R}=0.01$, ${\bar{\theta }}_{0}=0$. Similarly, the critical contraction coefficient $C_{0}$ for the supercritical Hopf bifurcation is found to be approximately 0.323, and the LCE drill remains static when $C_{0}\leq 0.323$. For $C_{0}=0.33,0.335,0.34$, self-rotation can be triggered, and their limit cycles are shown in Fig. 8 (a). Fig. 8 (b) displays the effects of contraction coefficient on the self-rotating frequency and amplitude. As $C_{0}$ increases, both the frequency and amplitude of self-rotation tend to increase. Eq. (9) provides evidence that increasing the contraction coefficient will cause an increase in light-driven contraction, which corresponds to an increase in absorbed light energy. This implies that increasing the contraction coefficient of LCE material can enhance the effective conversion of light energy to mechanical energy.

### 4.3 Influence of damping coefficient

**Fig. 9:**
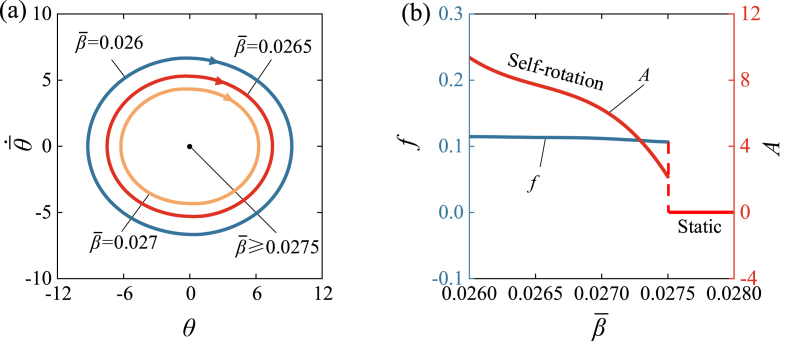
The influence of damping coefficient on the self-rotation, for ${\bar{I}}_{0}=1.9$, $\bar{k}=4600$, ${\overset{\cdot }{\bar{\theta }}}_{0}=5$, $C_{0}=0.34$, $\bar{R}=0.01$, ${\bar{\theta }}_{0}=0$. (a) Limit cycles. (b) Frequency and amplitude. There is a critical $\bar{\beta }\approx 0.0275$ for the supercritical Hopf bifurcation between the static regime and the self-rotation regime. The increase of $\bar{\beta }$ results in the declines of both the frequency and amplitude of self-rotation.

Fig. 9 and Video 4 show the influence of the damping coefficient $\bar{\beta }$ on self-rotation of LCE drill. Fig. 9 (a) illustrates limit cycles for different damping coefficients, while Fig. 9 (b) expresses the amplitude and frequency as functions of damping coefficient $\bar{\beta }$. In the calculation, we set ${\bar{I}}_{0}=1.9$, $\bar{k}=4600$, ${\overset{\cdot }{\bar{\theta }}}_{0}=5$, $C_{0}=0.34$, $\bar{R}=0.01$, ${\bar{\theta }}_{0}=0$. There exists a critical value of damping coefficient $\bar{\beta }$ approximately equal to 0.0275 for the supercritical Hopf bifurcation. When $\bar{\beta }\geq 0.0275$, the energy dissipation due to damping is too high and the energy input from the external environment is not sufficient to compensate for this dissipation, resulting in a static regime. However, for $\bar{\beta }=0.027$, $\bar{\beta }=0.0265$, $\bar{\beta }=0.026$, the self-rotation can be triggered. An increase in the damping coefficient leads to decreases both in the frequency and amplitude of the self-rotation. According to the derivation process in this article, the larger the damping coefficient, the more energy dissipation occurs, resulting in smaller amplitude and longer duration of the self-rotation in one single cycle.

### 4.4 Influence of spring constant

**Fig. 10:**
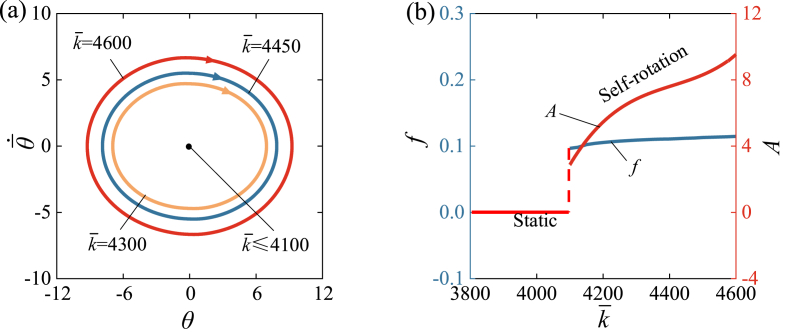
The influence of spring constant on the self-rotation, for $C_{0}=0.34$, $\bar{\beta }=0.026$, ${\bar{I}}_{0}=1.9$, ${\bar{\theta }}_{0}=0$, $\bar{R}=0.01$, ${\overset{\cdot }{\bar{\theta }}}_{0}=5$. (a) Limit cycles. (b) Frequency and amplitude. There is a critical $\bar{k}\approx 4100$ for the supercritical Hopf bifurcation between the static regime and the self-rotation regime. With the increase of $\bar{k}$, both the frequency and amplitude of self-rotation present increasing trends.

Fig. 10 and Video 5 show the influence of the spring constant $\bar{k}$ on the self-rotation of LCE drill. Fig. 10 (a) illustrates limit cycles for different spring constants, while Fig. 10 (b) represents the functional relationship between the spring constant $\bar{k}$, amplitude, and frequency. In the calculation, we set ${\bar{I}}_{0}=1.9$, $\bar{\beta }=0.026$, ${\overset{\cdot }{\bar{\theta }}}_{0}=5$, $C_{0}=0.34$, $\bar{R}=0.01$, ${\bar{\theta }}_{0}=0$. A critical value of spring constant $\bar{k}$ approximately equal to 4100 is present for the supercritical Hopf bifurcation. When $\bar{k}\leq 4100$, the lower tension of LCE fiber results in less net work done by the rotational moment, and therefore cannot compensate for the energy dissipated by damping to maintain self-rotation. Fig. 10 (b) shows the increasing trends of frequency and amplitude as the spring constant $\bar{k}$ increases. Similarly, this result can be understood from Eqs. (19), (21), (23), (24), in which $\bar{k}$ increases the rotational moment ${\bar{M}}_{F}$, leading to an increase in the net work done by the rotational moment in one cycle. Considering the physical meaning of $\bar{k}$, increasing the elastic modulus of LCE fiber can improve the efficiency of converting light energy to mechanical energy for engineering applications.

### 4.5 Influence of turnplate radius

**Fig. 11:**
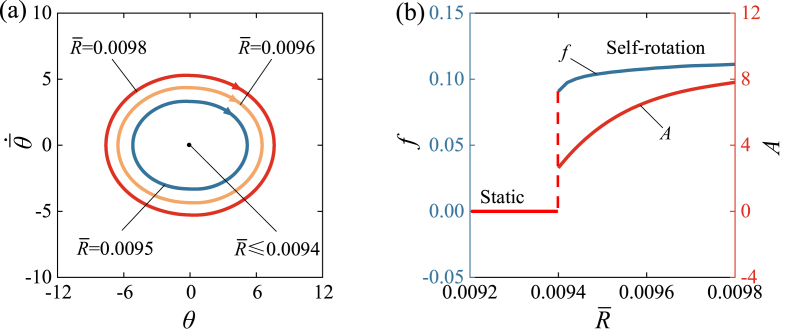
The influence of turnplate radius on the self-rotation, for $C_{0}=0.34$, $\bar{k}=4600$, ${\bar{I}}_{0}=1.9$, $\bar{\beta }=0.026$, ${\bar{\theta }}_{0}=0$, and ${\overset{\cdot }{\bar{\theta }}}_{0}=5$. (a) Limit cycles. (b) Frequency and amplitude. There is a critical $\bar{R}\approx 0.0094$ for the supercritical Hopf bifurcation between the static regime and the self-rotation regime. With the increase of $\bar{R}$, both the frequency and amplitude of self-rotation present increasing trends.

Fig. 11 and Video 6 show the influence of the turnplate radius $\bar{R}$ on the self-rotation of LCE drill. Fig. 11 (a) illustrates limit cycles for different spring constants, and Fig. 11 (b) represents the functional relationship between the radius $\bar{R}$, amplitude, and frequency. In the calculation, we set ${\bar{I}}_{0}=1.9$, $\bar{\beta }=0.026$, ${\overset{\cdot }{\bar{\theta }}}_{0}=5$, $C_{0}=0.34$, $\bar{k}=4600$, and ${\bar{\theta }}_{0}=0$. A critical value of radius $\bar{R}$ approximately equal to 0.0094 is present for the supercritical Hopf bifurcation. When $\bar{R}\leq 0.0094$, the lower tension of LCE fiber results in less net work done by the rotational moment, and therefore cannot compensate for the energy dissipated by damping to maintain self-rotation. Fig. 11 (b) shows the increasing trends of frequency and amplitude as the radius $\bar{R}$ increases. It is evident from Eq. (19) that the rotating moment ${\bar{M}}_{F}$ of turnplate increase with the increasing radius $\bar{R}$. This finding suggests that increasing the turnplate radius can improve self-rotation of the drill under steady illumination.

### 4.6 Influence of initial velocity

**Fig. 12:**
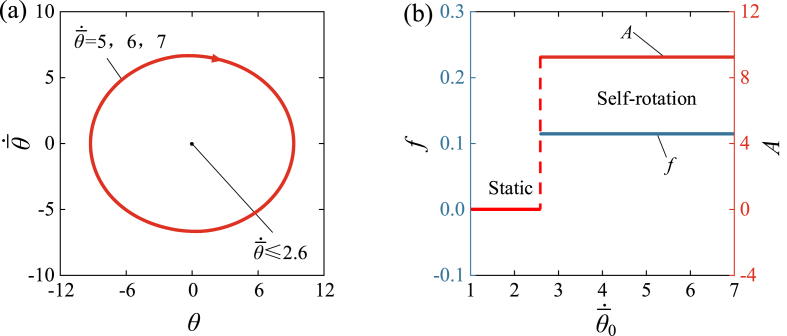
The influence of initial velocity on the self-rotation, for $C_{0}=0.34$, $\bar{k}=4600$, ${\bar{I}}_{0}=1.9$, $\bar{\beta }=0.026$, $\bar{R}=0.01$, ${\bar{\theta }}_{0}=0$. (a) Limit cycles. (b) Frequency and amplitude. A critical velocity ${\overset{\cdot }{\bar{\theta }}}_{0}\approx 2.6$ exists for triggering the self-rotation, and ${\overset{\cdot }{\bar{\theta }}}_{0}$ does not affect the amplitude and frequency of self-rotation.

Fig. 12 and Video 7 show the influence of initial velocity ${\overset{\cdot }{\bar{\theta }}}_{0}$ on the self-rotation of LCE drill. In the calculation, we set $C_{0}=0.34$, $\bar{k}=4600$, ${\bar{I}}_{0}=1.9$, $\bar{\beta }=0.026$, $\bar{R}=0.01$, ${\bar{\theta }}_{0}=0$. As can be seen from the graph, the initial velocity has no effect on the limit cycle, amplitude and frequency of the self-rotation. Fig. 12 (a) shows the limit cycle of self-rotation with different initial velocities. A critical velocity ${\overset{\cdot }{\bar{\theta }}}_{0}$ of approximately 2.6 exists in the phase transition between the static regime and the self-rotation regime. The self-rotation mechanism is triggered at velocities of 5, 6, 7, Fig. 12 (a) illustrates the limit cycles. Fig. 12 (b) shows the relationship between the frequency and amplitude of triggered self-rotation and initial velocity ${\overset{\cdot }{\bar{\theta }}}_{0}$. Neither frequency nor amplitude changes with the variation of ${\overset{\cdot }{\bar{\theta }}}_{0}$. Considering the equivalence of kinetic energy and potential energy in the conversion of ${\bar{\theta }}_{0}$ and ${\overset{\cdot }{\bar{\theta }}}_{0}$, the initial conditions always have no effect on the amplitude and frequency of self-rotation, which is an inherent property of self-oscillation [36].

## 5 Conclusions

Self-oscillating systems can continuously convert environment energy into mechanical work, and designing more self-oscillating systems lays the foundation for its widespread use in engineering. Inspired by the conventional hand drill, we have developed a novel self-rotating drill consisting of a LCE fiber and a turnplate under steady illumination. Based on dynamic LCE model, we propose a theoretical framework of the LCE drill under steady illumination to investigate the self-oscillating behaviors. Numerical calculations show that the LCE drill can undergo a supercritical Hopf bifurcation between the static regime and the self-rotation regime. The self-rotation of the LCE drill is interpreted as the light-driven contraction of the LCE fiber during the winding state under illumination. Due to the competition between light energy and damping dissipation, it manifests itself in a continuous periodic manner.

Furthermore, the frequency and amplitude of self-rotation depend primarily on several system parameters. Increasing the system parameters, including $\bar{R}$, $C_{0}$, ${\bar{I}}_{0}$, $\bar{k}$, can improve the self-rotating frequency and amplitude. On the other hand, an increase in $\bar{\beta }$ leads to decreases in both self-rotating frequency and amplitude. These qualitative behaviors of the self-rotation of LCE drill revealed by the model in this article is consistent with physical intuition. In practical scenarios, various complex factors can contribute to quantitative deviations from the actual conditions. These factors include the inertia of LCE fiber, the nonlinear constitutive law of fiber, their viscoelastic properties, bending stiffness, as well as the friction between the fiber and the turnplate. Hence, it is important to account for these factors in order to accurately capture the real-world behavior and performance of the system. For demonstration in future experiments, it is recommended to reduce the diameter of the LCE fiber, reduce its mass and viscoelasticity, and increase its reaction rate. In addition, during the self-rotating process, one should control the elongation of the LCE fiber to make that it is always in the linear elastic range and reduce the friction between the LCE fiber and the turntable.

It is worthwhile to further validate the self-rotation behaviors by designing the prototype of the LCE drill in the next work. To diminish the deviations of experimental results from the ideal theoretical situation, the LCE fiber in the experimentation should be less than 0.1 mm to reduce its own inertia and bending stiffness [76]. Meanwhile, it is recommended that the LCE fiber with a large contraction coefficient is fabricated and a large light intensity is utilized in experiments. In addition, the friction of the turnplate should be very small to decrease the damping dissipation for triggering the self-rotation. The self-rotating LCE drill proposed in this paper presents superiority in terms of simple and lightweight structure, customizable dimension, and fast response speed, and thus facilitates the design of compact and integrated systems, enhancing their applicability in microdevices and systems. This bears great significance in fields like micro-robotics, micro-sensors, and medical instruments, enabling the realization of smaller and higher-performance devices.

## CRediT authorship contribution statement

**Yong Yu:** Writing – original draft, Validation, Software, Methodology, Data curation, Conceptualization. **Haoyu Hu:** Writing – original draft, Validation, Software. **Haiyang Wu:** Validation. **Yuntong Dai:** Writing – review & editing, Validation. **Kai Li:** Writing – review & editing, Supervision, Investigation, Conceptualization.

## Declaration of competing interest

The authors declared that they have no conflicts of interest to this work. We declare that we do not have any commercial or associative interest that represents a conflict of interest in connection with the submitted manuscript entitled “A light-powered self-rotating liquid crystal elastomer drill”.
